# A machine learning approach to predict resilience and sickness absence in the healthcare workforce during the COVID-19 pandemic

**DOI:** 10.1038/s41598-022-12107-6

**Published:** 2022-05-16

**Authors:** Johannes Lieslehto, Noora Rantanen, Lotta-Maria A. H. Oksanen, Sampo A. Oksanen, Anne Kivimäki, Susanna Paju, Milla Pietiäinen, Laura Lahdentausta, Pirkko Pussinen, Veli-Jukka Anttila, Lasse Lehtonen, Tea Lallukka, Ahmed Geneid, Enni Sanmark

**Affiliations:** 1 Niuvanniemi Hospital, University of Eastern Finland, Kuopio, Finland; 2 Faculty of Medicine, University of Helsinki, Helsinki, Finland; 3 Clinical Research Institute HUCH, Helsinki University Hospital, Helsinki, Finland; 4 Department of Otorhinolaryngology and Phoniatrics—Head and Neck Surgery, Helsinki University Hospital, Helsinki, Finland; 5 Nordic Healthcare Group, Helsinki, Finland; 6 School of Business, Aalto University, Espoo, Finland; 7 Department of Oral and Maxillofacial Diseases, University of Helsinki and Helsinki University Hospital, Helsinki, Finland; 8 HUS Inflammation Center, Helsinki University Hospital, Helsinki, Finland; 9 HUS Diagnostic Center, HUSLAB, Helsinki University Hospital, Helsinki, Finland; 10 Department of Public Health, University of Helsinki, Helsinki, Finland

**Keywords:** Occupational health, Computational science

## Abstract

During the COVID-19 pandemic, healthcare workers (HCWs) have faced unprecedented workloads and personal health risks leading to mental disorders and surges in sickness absence. Previous work has shown that interindividual differences in psychological resilience might explain why only some individuals are vulnerable to these consequences. However, no prognostic tools to predict individual HCW resilience during the pandemic have been developed. We deployed machine learning (ML) to predict psychological resilience during the pandemic. The models were trained in HCWs of the largest Finnish hospital, Helsinki University Hospital (HUS, N = 487), with a six-month follow-up, and prognostic generalizability was evaluated in two independent HCW validation samples (Social and Health Services in Kymenlaakso: Kymsote, N = 77 and the City of Helsinki, N = 322) with similar follow-ups never used for training the models. Using the most predictive items to predict future psychological resilience resulted in a balanced accuracy (BAC) of 72.7–74.3% in the HUS sample. Similar performances (BAC = 67–77%) were observed in the two independent validation samples. The models' predictions translated to a high probability of sickness absence during the pandemic. Our results provide the first evidence that ML techniques could be harnessed for the early detection of COVID-19-related distress among HCWs, thereby providing an avenue for potential targeted interventions.

## Introduction

The coronavirus disease (COVID-19) pandemic has severely burdened healthcare systems worldwide. As a result, healthcare workers (HCWs) face unprecedented workloads and personal health risks leading to psychological distress^1^. Consequently, during the pandemic, frontline HCWs have been suffering from insomnia, anxiety, depression, and posttraumatic stress disorders in an increasing number compared to nonmedical staff^2–4^. Additionally, previous research has shown a surge in sickness absence during the pandemic compared to the time before the pandemic^5^, potentially threatening an individual hospital's functioning. In Finland, among those working in healthcare, an apparent increase in sickness absence days between 2019 and 2020 has been reported (https://www.tyoelamatieto.fi/en/articles/analysisKunta10SickLeave).

However, it has remained unclear why only some individuals are vulnerable to COVID-19-related distress and have a high risk of sickness absence, while others remain resilient throughout the pandemic^6^. Psychological resilience is often defined as the capacity to adapt and thrive while encountering challenging and stressful environments^7^. Given the interindividual differences in psychological resilience to adversities, individuals with poor resilience may be more vulnerable to the aforementioned consequences during the pandemic than those with higher resilience^8^. In a demanding work environment, resilient employees are less likely to be absent than employees with poor resilience^8^. In this light, identifying those with poor resilience during the pandemic is essential from the perspective of primary prevention and early intervention.

Previous group-level studies have outlined many potential predictors, such as particular medical professions, workplace status, psychological factors, and several sociodemographic factors, that predict poor resilience and COVID-19 outbreak-related mental health disorders^2,9–11^. However, these findings have not resulted in advances in the individual-level characterization of vulnerable individuals. Specifically, it has not been resolved whether any of these variables translate to predictions at the level of an individual HCW and whether predictions made in one hospital generalize to another hospital or another phase of the pandemic. Additionally, to the best of our knowledge, there are no tools to evaluate whether individual HCWs are on a trajectory to a high number of sickness absences during the pandemic.

Compared to group-level studies, machine learning (ML) techniques are applicable for deploying an individual-level predictive model based on many potential predictors^12^. During the pandemic, ML techniques have been harnessed to predict COVID-19 infection based on symptoms and imaging findings with very high accuracy^13,14^. Nevertheless, while a few efforts to predict HCWs' mental health using ML at a particular timepoint of the pandemic have been made^15,16^, no previous work has used ML to identify HCWs who remain poorly resilient to COVID-19-related distress throughout the pandemic. Previous work has shown that local increases in incidence rates of COVID-19 cases are reflected in psychological distress and sleeping problems in HCWs^17^. In this light, a prognostic tool that predicts resilience during the COVID-19 pandemic could be helpful, as it would enable the detection of individuals with poor resilience prior to the manifestation of potential mental disorder or sickness absence.

The overarching aim of the present work was to characterize a clinically manageable battery of items for which deployment with ML can reliably identify HCWs who are on a trajectory of poor resilience throughout the pandemic. We focused on two subsets of this broad concept, namely, "immunity" (i.e., remaining undisturbed during adversities) and "bouncing back" (i.e., return to former preadversity state)^18^. We trained our ML models in HCWs of the most prominent Finnish university hospital and evaluated the models' prognostic generalisability in two independent validation samples with similar follow-ups never used for training the models. Last, given that the COVID-19 pandemic has resulted in a surge in sickness absences^5^, we tested whether the applications of our ML models can be extended to identify individuals at high risk for sickness absence over the pandemic.

## Results

### Sociodemographic and clinical characteristics

The sociodemographic variables for the HUS discovery sample (participants from the Hospital District of Helsinki and Uusimaa), Kymsote (healthcare district of Kymenlaakso province) validation sample, and City of Helsinki validation sample are shown in Table 1. The three samples differed in terms of COVID-19 risk group members, BMI, number of smokers, number of physicians, and number of HCWs directly treating COVID-19 patients (Table 1). These differences were driven by a higher BMI and higher number of COVID-19 risk group members and smokers in Kymsote and a higher number of physicians and HCWs in direct contact with COVID-19 patients in HUS. To account for potential selection bias, we tested differences in demographic variables between HCWs of the HUS sample who participated (vs. did not participate) in the follow-up or the endpoint. We found that HCWs who participated in the follow-up or the endpoint were older (*t*(803) = 5.53, P-value < 0.001), more likely to be women (χ^2^ = 4.42, P-value = 0.035), and more likely to be physicians (χ^2^ = 6.40, P-value = 0.011). There were, however, no differences in BMI, number of COVID-19 risk group members, number of smokers, alcohol usage, or number of individuals in direct contact with COVID-19 patients.

**Table 1:** Sociodemographic characteristics of the included datasets.

	HUS (discovery set, N = 487)	KYMSOTE (validation set, N = 77)	Helsinki City (validation set, N = 322)	F-test/χ^2^ (P-value)
Age mean (SD)	44.2 (10.8)	44.9 (9.6)	44.8 (11.9)	0.40 (0.673)
Females N (%)	444 (91.1)	68 (88.3)	290 (90.1)	0.76 (0.685)
COVID-19 risk group N (%)	29 (6.0)	10 (13.0)	17 (5.3)	6.48 (0.039)
BMI mean(SD)	26.4 (5.1)	28.3 (6.0)	26.28645 (5.6)	3.69 (0.025)
Smokers N (%)	44 (9.0)	15 (19.5)	41 (12.7)	8.30 (0.016)
Usage of alcohol* N (%)	329 (67.6)	48 (62.3)	200 (62.1)	2.82 (0.24)
Physicians N (%)	111 (22.8)	9 (11.7)	42 (13.0)	14.79 (0.0006)
Direct contact to COVID-19 patients N (%)	202 (41.5)	15 (19.5)	86 (26.7)	26.91 (< 0.0001)

*Any weekly dose of alcohol.

**Figure 1:**
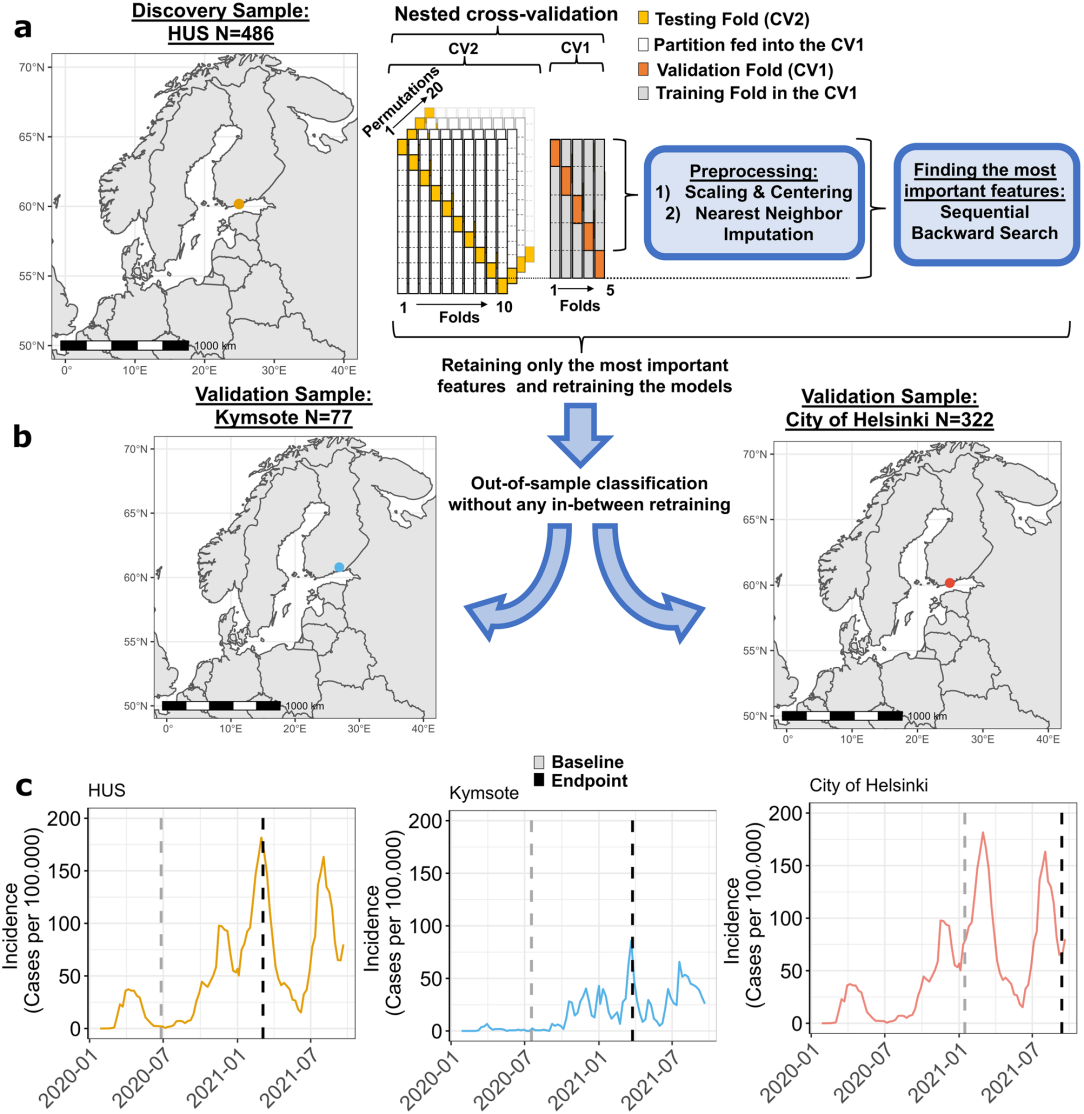
Flowchart depicting the analyses and samples of the present study. (**a**) Machine learning pipeline in the HUS discovery sample. (**b**) Condensed models (i.e., models trained on the most important variables) were applied to Kymsote and the City of Helsinki validation samples without any in-between retraining. (**c**) Incidence of COVID-19 in the district of each sample (data from https://sampo.thl.fi/pivot/prod/en/epirapo/covid19case/fact_epirapo_covid19case). Dashed lines represent the follow-up period of each sample. The models were trained to predict resilience over the follow-up based on each sample's baseline data (the gray dashed line). The endpoint is shown in black. Note that the incidence data for HUS and the City of Helsinki are identical since the HCWs of these datasets were working in the same area. Maps were created with "rnaturalearth" R package version 0.1.0.

The incidence of COVID-19 cases across the HUS and Kymsote regions is shown in Fig. 1c). Note that HCWs of the HUS and the City of Helsinki sample were working in the same area, as Helsinki is the most populated city in the HUS region. During the first wave of the epidemic in Finland, between the beginning of March 2020 and mid-June 2020, the COVID-19 incidence in the HUS region was approximately 309 cases/100 000 residents, and in the Kymsote region, it was approximately 29 cases/100 000 residents. After the number of cases started to continuously rise again in the autumn of 2020, between mid-December 2020 and mid-March 2021, the incidence of COVID-19 cases in the HUS region was approximately 1249 cases/100 000 residents, and in the Kymsote region, it was approximately 298 cases/100 000 residents. The point prevalence of COVID-19 cases differed between the HUS endpoint (point prevalence per 100 000 COVID-19 cases 182) and Kymsote endpoint (point prevalence per 100 000 COVID-19 cases 86): χ^2^ = 79.30, P-value < 0.0001. The incidence in the HUS region between mid-March 2021 and mid-June 2021, when the City of Helsinki follow-up surveys were still running, was approximately 856 cases/100 000 residents. For the same period in spring 2021, the incidence in Helsinki was approximately 1026 cases/100 000 residents^19^.

### Machine learning classification results

**Figure 2:**
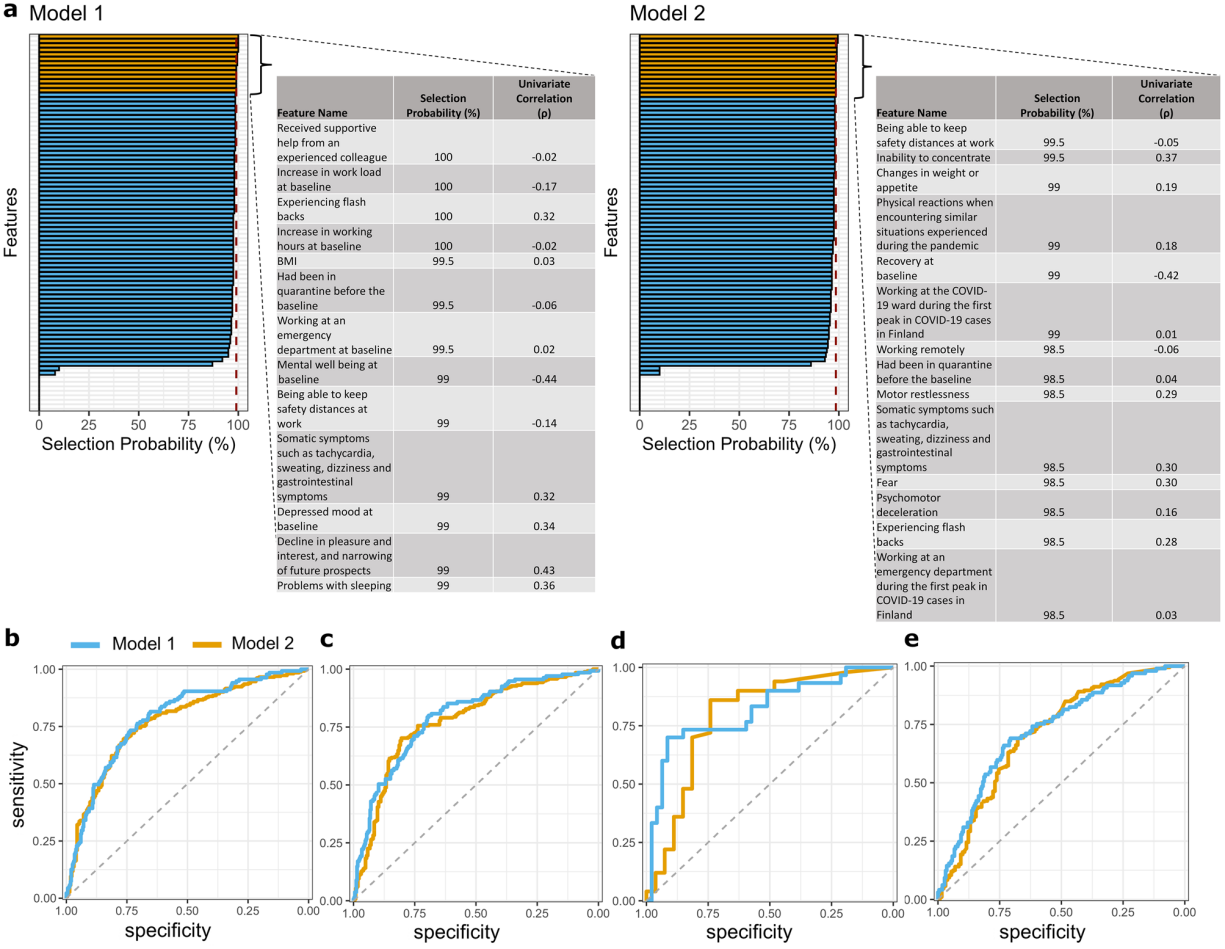
(**a**) Composition of predictive variable sets selected by Model 1 ("immunity") and Model 2 ("bouncing back"). The most predictive features are in yellow. (**b**) The performance of the full models in the HUS. The performance of the condensed models in (**c**) HUS, (**d**) Kymsote, and (**e**) The City of Helsinki sample.

The selection probabilities of each variable by the sequential backward search (SBS) wrapper^20^ using support vector machine (SVM) in the HUS discovery sample are shown in Fig. 2a. Self-reported psychiatric symptoms and work-related variables had the highest probability of being selected by both models. Using the top percentile selection criterion resulted in 13 variables (Model 1) and 14 variables (Model 2) used to train the corresponding condensed models. Four features ("Being able to keep safety distances at work", "Had been in quarantine before the baseline", "Somatic symptoms", and "Experiencing flashbacks") were on top percentiles and were thereby used in both condensed models. Univariate relationships between baseline variables and the six-month follow-up outcomes ranged between ρ = –0.44 and 0.43 (Model 1) and ρ = –0.42 and 0.37 (Model 2) in the HUS sample (Fig. 2a).

ROC curves depicting the classification performances across the three samples are shown in Fig. 2b–e. A detailed description of the classification results is provided in Supplementary Table 2. Classification of poor resilience using Model 1 in the HUS sample resulted in a balanced accuracy (BAC) of 73.1% and BAC of 72.4% for Model 2. The corresponding overall gains in certainty were 39.3 (Model 1) and 44.6 (Model 2). The respective condensed models (i.e., restricted to the top percentiles of the predictive baseline features) resulted in 72.7% and 74.3% for Model 1 and Model 2, respectively.

When the two HUS-trained condensed models were applied to the Kymsote sample, we found BACs of 77.1% and 71.7% for Model 1 and Model 2, respectively, with PSIs of 53.7 (Model 1) and 39.8 (Model 2). In the City of Helsinki sample, Model 1 resulted in a BAC of 67.27% (PSI = 29.3, AUC = 0.727, P-value < 0.0001) and Model 2 a BAC of 67.0% (PSI = 34.65, AUC = 0.714, P-value < 0.0001). Using the two condensed models, we found that across the three datasets, the number needed to predict ranged between 1.8–3.4 individuals.

### The relationships between the predictions of the two models and sickness absence

**Figure 3:**
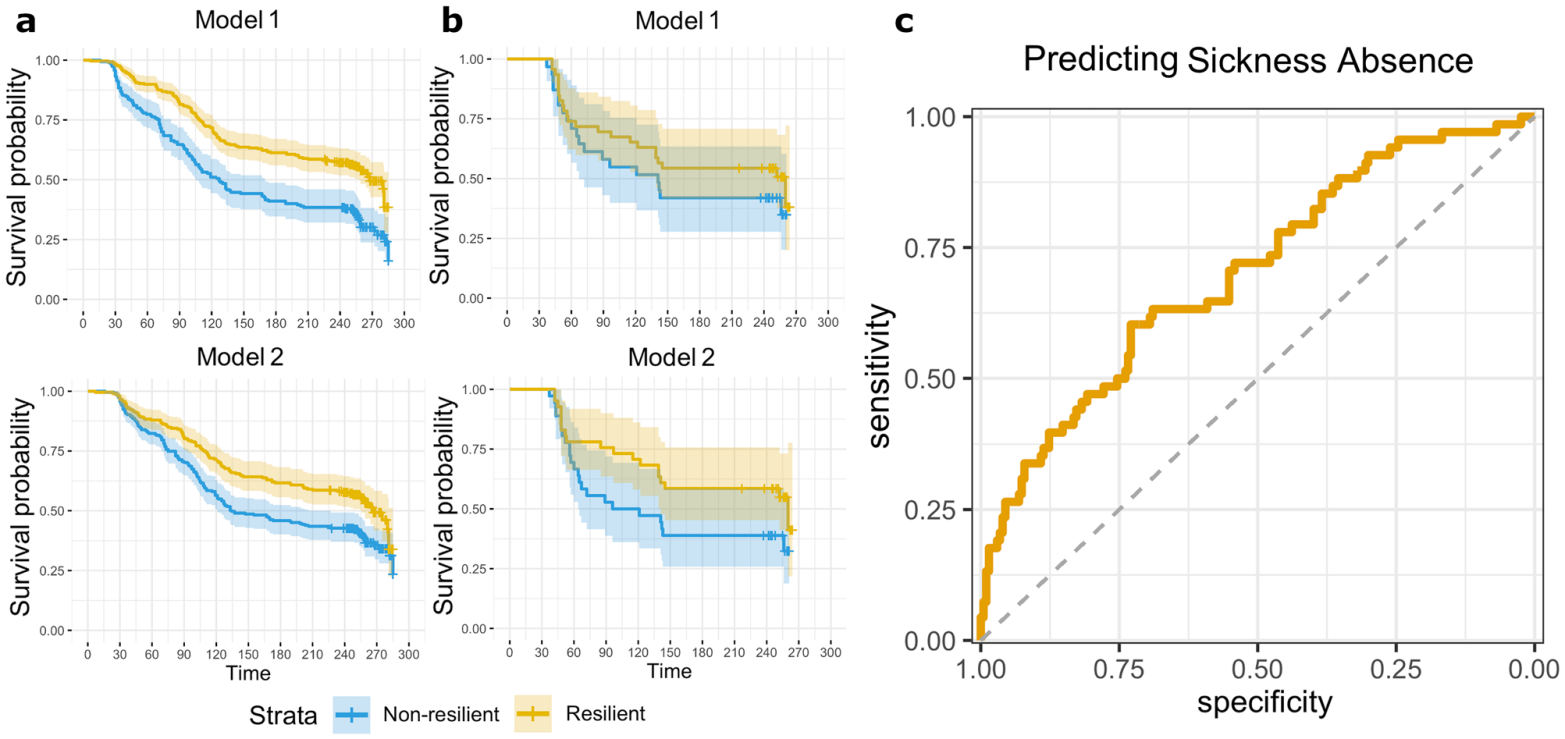
Relationships of the two ML models' predictions with sickness absences over the pandemic. Survival curves for sickness absences over the follow-up using Model 1 (top) and Model 2 (bottom) in (**a**) the HUS and (**b**) the Kymsote sample in HCWs predicted as nonresilient (blue) and resilient (yellow). (**c**) Predicting individuals whose sickness absences totaled over two weeks over the pandemic (between March 2020 and September 2021) using average predictions of the two models in the City of Helsinki sample.

Kaplan–Meier curves are presented in Fig. 3. In the HUS sample, log-rank analyses revealed higher rates of sick leaves in HCWs with nonresilience prognoses by Model 1 (χ^2^ = 25.5; P-value < 0.0001) and Model 2 (χ^2^ = 11.5; P-value = 0.0007). In the Kymsote sample, we found similar but nonsignificant effects: Model 1 (χ^2^ = 1.04; P-value = 0.31) and Model 2 (χ^2^ = 2.90; P-value = 0.085). Classification of sickness absence throughout the pandemic using the average predictions of the two ML models measured a BAC of 63.4% (sensitivity = 72.1%, specificity = 54.7%) and AUCs = 0.705 (P < 0.0001) in the City of Helsinki sample. These predictions were not explained by whether HCWs of the City of Helsinki were risk group members for COVID-19 or whether they were vaccinated for COVID-19 (all P-values > 0.1).

## Discussion

To our knowledge, this is the first study to evaluate the feasibility of predicting resilience in HCWs over the course of the COVID-19 pandemic using ML. Our findings provide crucial evidence that a stable trait of resilience during the future aggravation of the pandemic can be identified beforehand using models trained during the first year of the pandemic, including a relatively mild phase of the pandemic. We also found that individuals predicted as nonresilient demonstrated a higher probability of sickness absence over the pandemic than those identified as resilient. In the Kymsote sample, the relationship between the predicted resilience and sickness absence was similar to that of the other samples. However, a smaller sample size and more negligible incidence of local COVID-19 cases than the Helsinki region possibly rendered the findings statistically nonsignificant.

Highlighting the potential clinical translation of our results, our ML models based on a clinically manageable set of items provided similar prediction accuracies as the full panel of items and high generalisability to independent samples that had not been used for training the models. Using a data-driven wrapper technique for feature selection^20^, we were able to reduce the original number of variables into a clinically manageable set without a loss in prediction accuracy. Specifically, we found that a generalizable prognostic ML model based on just 13 baseline questions to predict future "immunity" and 14 to predict future "bouncing back" assessed at the stable phase of COVID-19 infections is feasible. The items selected by the two condensed models are mainly in line with previous COVID-19-related mental health literature in HCWs. Specifically, both models weighted work-related factors, primarily related to treating COVID-19 patients, frequently reported in previous literature^3,21–23^. Interestingly, both models used a safety distance-related question, supporting previous reports of severe concerns for contamination among HCWs^24^. Additionally, the two models selected previously reported factors related to good resilience throughout the COVID-19 pandemic, such as good working conditions and social support^25,26^. These factors underline the importance of the employer's role in strengthening resilience during the pandemic.

Our ML models demonstrated similar performance in Kymsote. However, we observed a slightly poorer performance in the City of Helsinki sample, which might partially stem from differences in the timing of the follow-up studies between the City of Helsinki and the other two samples. The HCWs of the City of Helsinki were followed in a later phase of the pandemic. It is, therefore, possible that some HCWs of the City of Helsinki had, up until that point, developed compensatory mechanisms to the pandemic, while other HCWs were experiencing ever-increasing pandemic-related distress. Additionally, HCWs of the City of Helsinki had been vaccinated during data collection, which might have positively affected the participants' mental state. Also, HCWs of the City of Helsinki dataset were mainly working at primary care, which might affect model generalization, as HCWs of the HUS discovery dataset were hospital and specialist medical care HCWs.

Although previous group-level work has elucidated the role of several factors (e.g., specific working position and old age^27^) on sick leaves among HCWs, no prior work on individual-level predictions on sickness absence over the pandemic in HCWs has been conducted. Using the predictions of the two models, we were able to predict those with a total of over two weeks of sickness absence through waves 1 to 5 of the pandemic (from March 2020 to October 2021) in the City of Helsinki sample at an acceptable accuracy (i.e., AUC > 0.7^28^). This finding is important from the point of potential prevention via targeted interventions, which would be essential given the observed surges in sickness absence over the pandemic^5^.

Since the reasons for sickness absence were unavailable, we can only speculate on the potential factors behind our findings. In line with previous epidemiological work, likely, the observed increment in sick leaves as a function of the pandemic course is partly due to an increment in infections and respiratory diseases^5^. The observed findings align with a previous report of a relationship between psychological stress and respiratory infections^29^, as those identified as nonresilient might be more vulnerable to pandemic-related stress. Specifically, nonresilient HCWs may have a more vulnerable immune system (vs. resilient HCWs), as there is already evidence that chronic stress can cause immunological changes^30^.

On the other hand, it is also likely that part of the observed increase in sickness absence is due to distress-related sickness absence, as previous studies have shown that HCWs have suffered from psychological distress and various mental health symptoms during the pandemic^1–3^. Healthcare work is often demanding, and working overtime among HCWs even before the pandemic was relatively common^31^. Consequently, an increment in excessive workload potentially results in increased sickness absence days^32^. During the pandemic, various nontargeted strategies, including organizational, personal, and interpersonal strategies, to improve resilience in HCWs at the system level have been discussed^11^. Although scarce evidence on the effectiveness of these interventions to improve resilience among HCWs during disease outbreaks exists today^33^, future studies with clinical settings should investigate whether ML-based targeted interventions (e.g., by employing cognitive-behavioral therapy or mindfulness-based interventions^34^) are viable.

Our study has several limitations. First, we used online survey data without any clinical assessment. Therefore, our analyses are limited by reliance on self-reported data. Second, our study had low participation and a relatively high drop-out rate in all three samples, thereby potentially compromising generalizability to nonparticipating subjects. For instance, our attrition analyses in the HUS discovery sample revealed that HCWs who did (vs. did not) participate in the follow-up were more likely older women and physicians. Third, we focused only on two dimensions of the broad concept of resilience^18,35,36^. Future work should extend to other dimensions (e.g., personal growth during the pandemic). Fourth, although our results were validated in two independent samples, future investigations in other countries are necessary to test our findings' generalizability further. It is possible that the features demonstrating the highest predictive performance in our datasets would not be as predictive in samples with a very different healthcare system. Additionally, future ML studies should investigate whether an increment in classification accuracy could be reached by incorporating other modalities (e.g., inflammatory, neuroimaging, cognitive, and genetic data) outside of the questionnaire battery. The utility of such an approach has been demonstrated in previous work on psychosis prediction in a European multisite study^37^.

In conclusion, our results provide the first evidence that ML techniques have the potential to identify poor resilience at the level of an individual HCW during the COVID-19 pandemic. Importantly, these predictions translated to a high probability of sickness absence over the pandemic. With further development, these models may potentially provide tools to enhance early detection and, thereby, provide an avenue for targeted primary prevention during sudden stressful phases of the pandemic. Future studies are required to determine whether these individualized predictions on resilience enhance the diagnosis of psychiatric disorders that have been associated with COVID-19-related distress.

## Methods

### Participants

The Ethical Committee of Helsinki University Hospital approved the present study (HUS/1450/2020), and the study participants of each of the three datasets gave written informed consent. All methods were performed in accordance with relevant guidelines and regulations and the Declaration of Helsinki. Supplementary Fig. 1 presents the collection of the three study samples. In this study, we included only participants at direct contact with the patients (nurses, physicians, midwives, laboratory technicians, radiographers, practical nurses, or paramedics, with a minimum age of 18 years, and employed in HUS or Kymsote since the beginning of the first wave, or in the City of Helsinki during the current pandemic), as healthcare professionals have shown a higher risk of developing psychiatric symptoms than nonclinical staff^3^. HCWs were informed about our study by mass email sent to all existing email addresses of HCWs in the studied organizations. Due to technical issues, some individuals not part of the intended target group may have also received the mass email, but we excluded participants who did not fulfill the study's inclusion criteria.

We used the participants from the Hospital District of Helsinki and Uusimaa (HUS, Helsinki University Hospital) as our model discovery dataset (Fig. 1a). In HUS, all 17 740 healthcare professionals were informed about the study by email, and finally, 862 eligible HCWs participated in the baseline survey during summer 2020 after the first wave of the pandemic in Finland. Of those participants, 487 completed the six-month follow-up consisting of regular monthly surveys. A similar contemporary follow-up study took place during the second wave in the healthcare district of Kymenlaakso Province (Kymsote), where 6155 healthcare and social services workers were informed about the study by a group email. Of 5625 Kymsote HCWs, 134 eligible participants took part at baseline in summer 2020, and 77 completed the six-month follow-up. The Kymsote sample was used as a validation sample for the deployed ML models. In both HUS and Kymsote samples, the baseline survey evaluated the period since the first wave in Finland in March 2020, as shown in Fig. 1c.

During the third and fourth waves of the epidemic, a follow-up study was also conducted among the personnel of the City of Helsinki, and 9306 healthcare, social services, and oral healthcare workers working mainly in primary care were invited to participate via group email forwarded to staff by their supervisors. In the City of Helsinki sample, the study was launched at a later pandemic stage (the end of January 2021), when COVID-19 vaccinations of HCWs had already been started. Of 7022 HCWs in the City of Helsinki, 600 HCWs participated in the baseline survey, and 322 participants completed the follow-up.

### Predictor variables and definition of poor resilience

We used an online survey consisting of questions covering participants’ sociodemographic information, mental health, leisure time, morbidities (belonging to a COVID-19 risk group), work and working environment with protection and safety measures in hospitals and other healthcare surroundings, and other questions related to COVID-19 available at the three datasets. The same questionnaire was sent to participants every 1–2 months, and the final follow-up survey was administered after 6 months. We used one-hot encoding for categorical features. Altogether, 84 baseline features were used to predict psychological distress over the six-month follow-up in healthcare workers available at each of the three datasets.

The ML models to predict future resilience in the present study were trained using the HUS sample at baseline when the number of COVID-19 cases was low. Nevertheless, the baseline survey evaluated the period since the beginning of the first wave in Finland (Fig. 1c). In the present study, we focused on two often investigated dimensions of resilience in previous COVID-19-related studies^6,24,38^, namely, (1) psychological immunity to adverse events (Model 1) and (2) bouncing back to equilibrium (Model 2). Specifically, we trained Model 1 on baseline variables in the HUS sample to identify HCWs reporting mental well-being below normal at ≥ 50% at the subsequent timepoints (i.e., not including baseline) over the six-month follow-up. Similarly, we trained Model 2 on those who reported insufficient recovery from clinical work during the pandemic at ≥ 50% subsequent follow-up timepoints. Although recovery in itself does not equal resilience, we justified this definition, as working during the pandemic can be viewed as a transient stressor of which repetitive recovery to a pre-pandemic state is only possible for sufficiently resilient individuals^18^. We included HCWs with both baseline and endpoint data and at least one follow-up timepoint available in our analyses.

### Machine learning pipeline

The two ML models were trained using R version 4.0.3 (https://cran.r-project.org) accompanied by "e1071"^39^ wrapped in the "mlr"^40^ package. First, using the HUS sample, we trained the two ML models on all baseline features using repeated nested cross-validation to prevent information leakage between training and testing and to provide unbiased predictive generalisability (Fig. 1a). In the inner loop of cross-validation (CV1), training data were scaled and centered at zero, and the missing variables were imputed with the nearest neighbor method. Next, each preprocessed training sample was entered into a sequential backward search (SBS) wrapper using a support vector machine (SVM) to iteratively drop redundant features, of which noninclusion improved the model performance. The search of the SBS was stopped if there were no features left of which noninclusion improves the performance of the SVM. We chose SVM over other algorithms due to its wide usage in previous psychiatric ML studies^12^. We used maximal balanced accuracy (BAC; i.e., average sensitivity and specificity) as a criterion for winning models across the range of SVM hyperparameters (details in Supplementary Table 1) optimized using a random search with 100 iterations. Finally, we applied the winning models of CV1 to the respective out-of-training test folds (CV2).

Second, using each features’ probability of being selected by the SBS wrapper, we retrained the two models using only the highest performing features (i.e., the top one percent in the SBS selections, Fig. 1b) to reduce the original feature space into a manageable set of items. Using this reduced feature space, we used the same setup of repeated, nested cross-validation as in the above classifications to train the models to predict the same two resilience outcomes as above. Last, we applied these two condensed models, without any in-between retraining, to Kymsote and the City of Helsinki samples (Fig. 1c).

### Statistical analyses

We evaluated the regional COVID-19 prevalence and incidences from open data provided by the Finnish Institute for Health and Welfare^19^ to assess the local disease burden of studied regions throughout the epidemic. We used the following R packages for the statistical analyses: "pROC"^41^, "ggplot2"^42^, "ggfortify", and "survival"^43^ packages. Maps in Fig. 1. were created using “rnaturalearth”^44^ package. We compared the demographic characteristics between the three datasets using the T-test, χ^2^-test, and Fisher's exact test as specified in the Results section. Using Spearman's correlation, we calculated univariate correlations between the baseline variables and the two resilience outcomes.

We assessed the prognostic gains of each ML model using the prognostic summary index (PSI), which describes gain in certainty compared to the pretest probabilities in the test population over the subsequent timepoints^45^. We also assessed the number needed to predict, describing a number of persons with poor resilience who need to be examined to correctly detect one person with poor resilience^45^. The significance of the predictive performance of a given model was determined with nonparametric permutation-based tests of the observed AUC for predicting the outcome using the model's predictions compared to an empirical null distribution created by resampling a given outcome label 5,000 times.

Last, we assessed whether the individuals predicted as resilient by the two ML models would demonstrate fewer sickness absence days over the follow-up compared to those prognosed as poorly resilient. For this purpose, we first used Kaplan–Meier analyses with the log-rank test in the HUS and Kymsote datasets. Only the total number of sickness absences throughout the pandemic (assessed at the endpoint questionnaire) in the City of Helsinki sample was available. Using the predictions of the two ML models in the City of Helsinki sample, we investigated whether the average prediction of the two models can identify those who have total sickness absences of over two weeks over the whole course of the pandemic. This analysis was restricted to those who had not been placed in quarantine at baseline to account for quarantine-related selection bias in sickness absence.

## Supplementary Information

Supplementary Information 1.

Supplementary Information 2.

Supplementary Information 3.

## Data Availability

The datasets analyzed in this study are not publicly available due to participant privacy and security concerns. Researchers can apply access for these data from the last author (enni.sanmark@hus.fi) upon reasonable request.

## Code availability

The codes to reproduce the present study's findings are available from the corresponding author upon reasonable request.
