# Supplementary material for: A machine learning approach to predict resilience and sickness absence in the healthcare workforce during the COVID-19 pandemic
Source: Sci Rep. 2022 May 16;12:8055. doi: 10.1038/s41598-022-12107-6 (PMC9109448; doi:10.1038/s41598-022-12107-6)
Supplement: Supplementary file 1 — Supplementary Information 1. [file 41598_2022_12107_MOESM1_ESM.docx]

**Supplementary Table 1.** The SVM hyperparameters.

| Hyperparameter | Values |
| --- | --- |
| Kernels | Polynomial, Radial, Sigmoid |
| Degree | 1,2,3 |
| Gamma | 0,1 ... 10 |
| Cost | 2^-8^... 2^8^ |
