# Supplementary material for: A machine learning approach to predict resilience and sickness absence in the healthcare workforce during the COVID-19 pandemic
Source: Sci Rep. 2022 May 16;12:8055. doi: 10.1038/s41598-022-12107-6 (PMC9109448; doi:10.1038/s41598-022-12107-6)
Supplement: Supplementary file 2 — Supplementary Information 2. [file 41598_2022_12107_MOESM2_ESM.docx]

**Supplementary Table 2.** Predictive performances of the two models in the three study samples.

|  | True Positive | True Negative | False Positive | False Negative | Balanced Accuracy % | Sensitivity % | Specificity % | Positive predictive value % | Negative predictive value % | Area Under Curve | Diagnostic Odds ratio | Predictive Summary Index | Number Needed to Predict |
| --- | --- | --- | --- | --- | --- | --- | --- | --- | --- | --- | --- | --- | --- |
| Model 1 (all features): HUS | 96 | 264 | 88 | 39 | 73.06 | 71.11 | 75.00 | 52.17 | 87.13 | 0.7870 | 7.384216 | 39.30 | 2.544529 |
| Model 2 (all features): HUS | 185 | 167 | 58 | 77 | 72.42 | 70.61 | 74.22 | 76.13 | 68.44 | 0.7726 | 6.916791 | 44.57 | 2.243662 |
| Condensed Model 1 (top features): HUS | 97 | 259 | 93 | 38 | 72.72 | 71.85 | 73.58 | 51.05 | 87.21 | 0.795 | 7.108457 | 38.26 | 2.613696 |
| Condensed Model 2 (top features): HUS | 196 | 166 | 59 | 66 | 74.29 | 74.81 | 73.78 | 76.86 | 71.55 | 0.777 | 8.356751 | 48.41 | 2.065689 |
| Condensed Model 1 (top features): KYMSOTE | 22 | 38 | 9 | 8 | 77.09 | 73.33 | 80.85 | 70.97 | 82.61 | 0.8156 | 11.60833 | 53.58 | 1.866368 |
| Condensed Model 2 (top features): KYMSOTE | 31 | 22 | 5 | 19 | 71.74 | 62.00 | 81.48 | 86.11 | 53.66 | 0.7937 | 7.178243 | 39.77 | 2.514458 |
| Condensed Model 1 (top features): Helsinki City | 68 | 145 | 80 | 29 | 67.27 | 70.10 | 64.44 | 45.95 | 83.33 | 0.7274 | 4.248549 | 29.28 | 3.415301 |
| Condensed Model 2 (top features): Helsinki City | 145 | 76 | 54 | 47 | 66.99 | 75.52 | 58.46 | 72.86 | 61.79 | 0.7141 | 4.341531 | 34.65 | 2.886003 |
