# Supplementary material for: A machine learning approach to predict resilience and sickness absence in the healthcare workforce during the COVID-19 pandemic
Source: Sci Rep. 2022 May 16;12:8055. doi: 10.1038/s41598-022-12107-6 (PMC9109448; doi:10.1038/s41598-022-12107-6)
Supplement: Supplementary file 3 — Supplementary Information 3. [file 41598_2022_12107_MOESM3_ESM.docx]

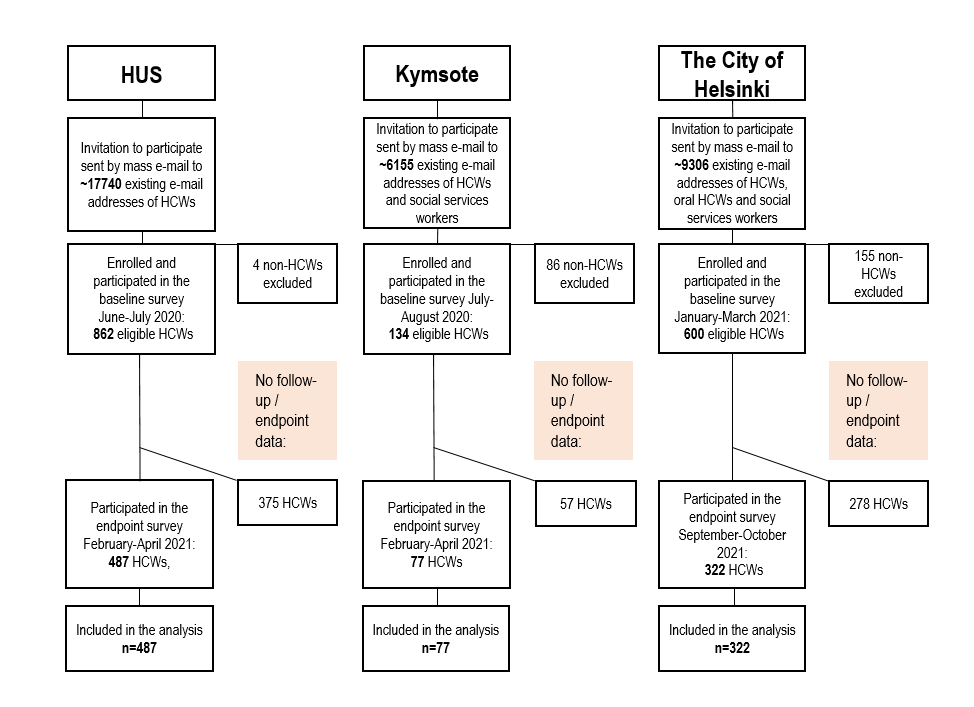


**Supplementary Figure 1.** Flow chart of study enrolment and participants. HCW, healthcare worker; HUS, Helsinki University Hospital; Kymsote, Social and Health Services in Kymenlaakso.
